# Relationships of sphenoid sinus pneumatization with internal carotid artery characteristics

**DOI:** 10.1371/journal.pone.0273545

**Published:** 2022-08-25

**Authors:** Yun Jin Kang, Jin-Hee Cho, Do Hyun Kim, Sung Won Kim

**Affiliations:** 1 Department of Otolaryngology-Head and Neck Surgery, Yeouido St. Mary’s Hospital, College of Medicine, The Catholic University of Korea, Seoul, Republic of Korea; 2 Department of Otolaryngology-Head and Neck Surgery, Seoul St. Mary’s hospital, College of Medicine, The Catholic University of Korea, Seoul, Republic of Korea; AIIMS: All India Institute of Medical Sciences, INDIA

## Abstract

**Objective:**

We explored the clinical significances of the relationships among sphenoid sinus aeration, intersphenoid sinus septum (ISS), and internal carotid artery (ICA).

**Methods:**

We retrospectively reviewed the preoperative paranasal sinus computed tomography scans and the medical charts of 490 patients who were treated by the endoscopic endonasal transsphenoidal approach. We analyzed sphenoid sinus pneumatization, number of ISS, and positional relationships between the ICA and ISS (including ICA prominence and the thickness of surrounding bone).

**Results:**

ISS were often present in the ICAs of patients with presellar pneumatization (36.2%; p = 0.042). Sphenoid sinus pneumatization status significantly differed according to number of ISS (p < 0.001), ICA prominence (p < 0.001), ISS insertion into the ICA (p = 0.042), and distance from ISS to ICA (p = 0.004). When sphenoid sinus aeration was poor, the ICA was not prominent, and the ISS were attached to or lay close to the paraclival ICA.

**Conclusions:**

Patients with presellar pneumatization exhibited less prominent ICAs, and more ISS attached to or near the paraclival ICA, than did other patients. Therefore, particular caution is required when using the endoscopic endonasal transsphenoidal approach to treat patients with poor sphenoid sinus aeration.

## Introduction

The pneumatization of the sphenoid sinus proceeds from about 3 years old to about 16 years old [1]. The sphenoid sinus is a structure that has many variations [2]; the degree of pneumatization differs from person to person, and according to the degree, it can be divided into conchal, presellar, sellar, and postsellar types [1,2]. In addition, the intersphenoid sinus septum (ISS) exists in various numbers and forms [3–5].

The internal carotid artery (ICA), opticocarotid recess (OCR), and optic nerve (ON) play important roles in confirming the structural orientation of the sphenoid sinus and sella floor [6–9]. The anatomical variances associated with ISS and pneumatization of the sphenoid sinus may affect the structural orientation when approaching the perisellar, clivus or pituitary gland lesion [5,10].

Various ISS forms obstruct the visibility of important structures such as ICA prominence and the OCR. Consequent failure to identify important nerves or blood vessels can result in damage and serious complications such as blindness or life-threatening blood loss [2,11]. Therefore, it is important to identify the details of the anatomy by preoperative paranasal sinus computed tomography (PNS CT) before commencing surgery using an endoscopic endonasal transsphenoidal approach [12–15].

Several studies have identified the pneumatization of the sphenoid sinus and ISS and the relationship with the surrounding ICA and ON [1,5,10,16,17]. However, to the best of our knowledge, none has comprehensively summarized the positional relationship between the ICA and the ISS and the degree of aeration of the sphenoid sinus in a large population. Therefore, in this study we investigated the relationship between sphenoid sinus type and the anatomical structure within the sinus to improve understanding of the ICA, which is an important structure in surgery using an endoscopic endonasal transsphenoidal approach.

## Materials and methods

This study enrolled 490 patients who underwent endoscopic endonasal transsphenoidal surgery at Seoul St. Mary’s Hospital (Catholic University of Korea, Seoul, South Korea) from February 2009 to July 2020. Medical chart records and preoperative paranasal sinus (PNS) computed tomography (CT) scans were retrospectively reviewed. Exclusion criteria were age < 18 years (such patients have incomplete anatomical structures), revision surgery cases, and cases in which ISS were not clearly observed because of large sellar lesions. The study protocol was approved by the institutional review committee of the Catholic University of Korea (approval no. KC17RESI0354). PNS CT images were axial projections with a thickness of 0.6 mm. Coronal and sagittal images were reconstructed using the same (non-enhanced) bone shadow settings. We focused on sphenoid sinus pneumatization status and the ISS; whether the ICA was prominent; the thickness of bone surrounding the ICA; and the distance from the origin of ISS to the ICA.

### Statistical analysis

The effect size of the ANOVA test to achieve 0.05 type 1 error and 80% power was calculated by G-Power (version 3.1.9.2; Heinrich-Heine-Universität Düsseldorf, Düsseldorf, Germany) [18]. For the incidence of ISS insertion into paraclival ICA, at least 51 patients per each group were required with assuming 26.6~63.1% per each group and 43.8% of total incidence [1].

All analyses were performed using R statistical software (R Foundation for Statistical Computing, Vienna, Austria). The significances of differences according to the extent of sphenoid aeration were evaluated by one-way analysis of variance, followed by post hoc analysis using Tukey’s honest significant difference test. Correlations between categorical variables were determined by Pearson correlation. A p-value < 0.05 was considered statistically significant.

### Classification of sphenoid sinus pneumatization

Pneumatization was graded according to the proportions of air and bone in the sphenoid sinus. On the sagittal plane, if the posterior end of pneumatization extended to the front of the sellae, the pneumatization was considered presellar; if the end lay between the anterior and posterior ends of the sellae, the pneumatization was considered sellar; and if the end lay beyond the posterior ends of the sellae, the pneumatization was considered postsellar. Minimal pneumatization was considered conchal [1,4,5].

### Classification of ISS

The ISS constituted an anatomical structure evident in axial scans of the sphenoid sinus; the ISS ran (completely or incompletely) from the posterior or lateral bony wall to the anterior wall.

### Characteristics of ICA

We focused on ICA prominence, surrounding bone thickness, and the distance between the origin of ISS and the ICA. As in a previous study [19], ICA prominence was graded according to whether the paraclival ICA protruded into the sphenoid sinus on PNS or whether it was embedded in the posterior or lateral bony wall CT. The thickness of bone surrounding the ICA was the thinnest region of bone that was exposed along with the sphenoid sinus [20]; it was graded as a bony dehiscence of < 0.5 mm, 0.5–1 mm, or > 1 mm. When ISS were not inserted into the paraclival ICA, the conditions were classified as follows: 1) ISS insertion points in the axial plane lay anterior to the paraclival ICA and covered the ICA; or 2) ISS insertion points in the axial plane lay behind the paraclival ICA and did not cover the ICA. The distance from the ISS insertion points to the ICA was classified as < 5 mm, 5–10 mm, or > 10 mm.

## Results

### Types of sphenoid sinus pneumatization, intersphenoid sinus septum and internal carotid artery features

The most common types of sphenoid sinus pneumatization were postsellar (47.9%) and sellar (45.7%). Sphenoid sinuses with ≥ 3 ISS were most common (53.2%). Table 1 summarizes the ISS number, paraclival ICA prominence, bone thickness around the ICA, ISS insertion into the paraclival ICA, and location of the paraclival ICA and its distance from the intersphenoid sinus, according to sphenoid sinus type. Presellar pneumatization was most commonly associated with two ISS, while sellar and postsellar pneumatizations were most commonly associated with > 3 ISS (p < 0.001).

**Table 1:** Relationships of sphenoid sinus pneumatization type with ISS and ICA features, as revealed by preoperative axial paranasal computed tomography*.

	Conchal (n = 2)	Presellar (n = 58)	Sellar (n = 430)	Postsellar (n = 450)	P-value
Age (years), mean ± standard deviation	60.0 ± 0.0	51.2 ± 16.3	51.5 ± 12.9	40.4 ± 15.2	< 0.001
ISS (complete, incomplete)					< 0.001
No ISS	2 (100.0%)	0 (0.0%)	0 (0.0%)	0 (0.0%)	
One IS septum	0 (0.0%)	6 (10.3%)	60 (14.0%)	38 (8.4%)	
Two ISS	0 (0.0%)	38 (65.5%)	162 (37.7%)	134 (29.8%)	
> 3 ISS	0 (0.0%)	14 (24.1%)	208 (48.4%)	278 (61.8%)	
ICA prominent	NA	10 (27.0%)	112 (36.2%)	157 (54.3%)	< 0.001
Bone thickness around the ICA (indicating exposure to sphenoid sinus)					0.253
< 0.5 mm	NA	8 (21.6%)	47 (15.2%)	48 (16.6%)	
0.5–1 mm	NA	11 (29.7%)	97 (31.4%)	103 (35.6%)	
1–5 mm	NA	13 (35.1%)	109 (35.3%)	102 (35.3%)	
5–10 mm	NA	1 (2.7%)	35 (11.3%)	16 (5.5%)	
> 10 mm	NA	4 (10.8%)	21 (6.8%)	20 (6.9%)	
Insertion of ISS into paraclival ICA	NA	21 (36.2%)	111 (25.8%)	151 (33.6%)	0.042
Location of ISS origin					0.052
Lateral to paraclival ICA	NA	26 (70.3%)	195 (63.1%)	209 (72.3%)	
Medial to paraclival ICA	NA	11 (29.7%)	114 (36.9%)	80 (27.7%)	
Distance from ISS origin to the ICA					0.004
< 0.5 mm	NA	5 (13.5%)	18 (5.8%)	4 (1.4%)	
0.5–1 mm	NA	0 (0.0%)	16 (5.2%)	14 (4.8%)	
1–5 mm	NA	18 (48.6%)	121 (39.2%)	109 (37.7%)	
5–10 mm	NA	9 (24.3%)	120 (38.8%)	117 (40.5%)	
> 10 mm	NA	5 (13.5%)	34 (11.0%)	45 (15.6%)	

Abbreviations: ICA, internal carotid artery; ISS, intersphenoid sinus septa; NA, not available.

*Data are shown as No (%) unless otherwise indicated.

Paraclival ICA prominence evident on axial PNS CT was most common in patients with postsellar pneumatization (p < 0.001). Bone thickness around the paraclival ICA was measured as the thinnest part of bone associated with the sphenoid sinus on axial PNS CT. In patients with presellar and sellar pneumatizations, the most common distance was 1–5 mm; in patients with postsellar pneumatizations, distances of 0.5–1 mm and 1–5 mm were frequently reported, although this trend was not statistically significant (p = 0.253). The frequency of ISS insertion into the paraclival ICA was significantly greater in patients with presellar pneumatizations (p = 0.042). We carefully analyzed patients in whom ISS were adjacent to the ICA, rather than inserted into the paraclival ICA. We measured the closest distances between ISS insertion sites and the paraclival ICA. In patients with presellar and sellar pneumatizations, most distances were 1–5 mm; in patients with postsellar pneumatizations, most distances were 5–10 mm (p = 0.004).

Post hoc analysis revealed that sphenoid sinus aeration significantly (all p < 0.01) affected ISS number, ICA prominence, ISS insertion into the ICA, and the distance between the ISS and ICA (except in patients with sellar and postsellar pneumatizations who had multiple ISS; p = 0.99).

### Relationship between sphenoid sinus pneumatization and other factors

Sphenoid sinus pneumatization was significantly correlated with ISS (r = 0.196, p < 0.001), ICA prominence (r = 0.145, p < 0.001), and the distance from the ISS insertion site to the ICA (r = 0.128, p = 0.01). An even stronger correlation was observed between sphenoid sinus pneumatization and age (r = –0.336, p < 0.001). No significant correlations of sphenoid sinus pneumatization were observed with the thickness of bone surrounding the paraclival ICA, ISS insertion into the ICA, or the relative locations of the ISS and ICA. However, strong correlations of ICA prominence with the thickness of bone surrounding the ICA were observed in the postsellar pneumatization group (r = –0.444, p < 0.001) and the presellar and sellar pneumatization groups (r = –0.450, p < 0.001); strong correlations of ICA prominence with ISS insertion into the ICA were observed in the presellar and sellar pneumatization groups (r = 0.419, p < 0.001).

## Discussion

**Fig 1:**
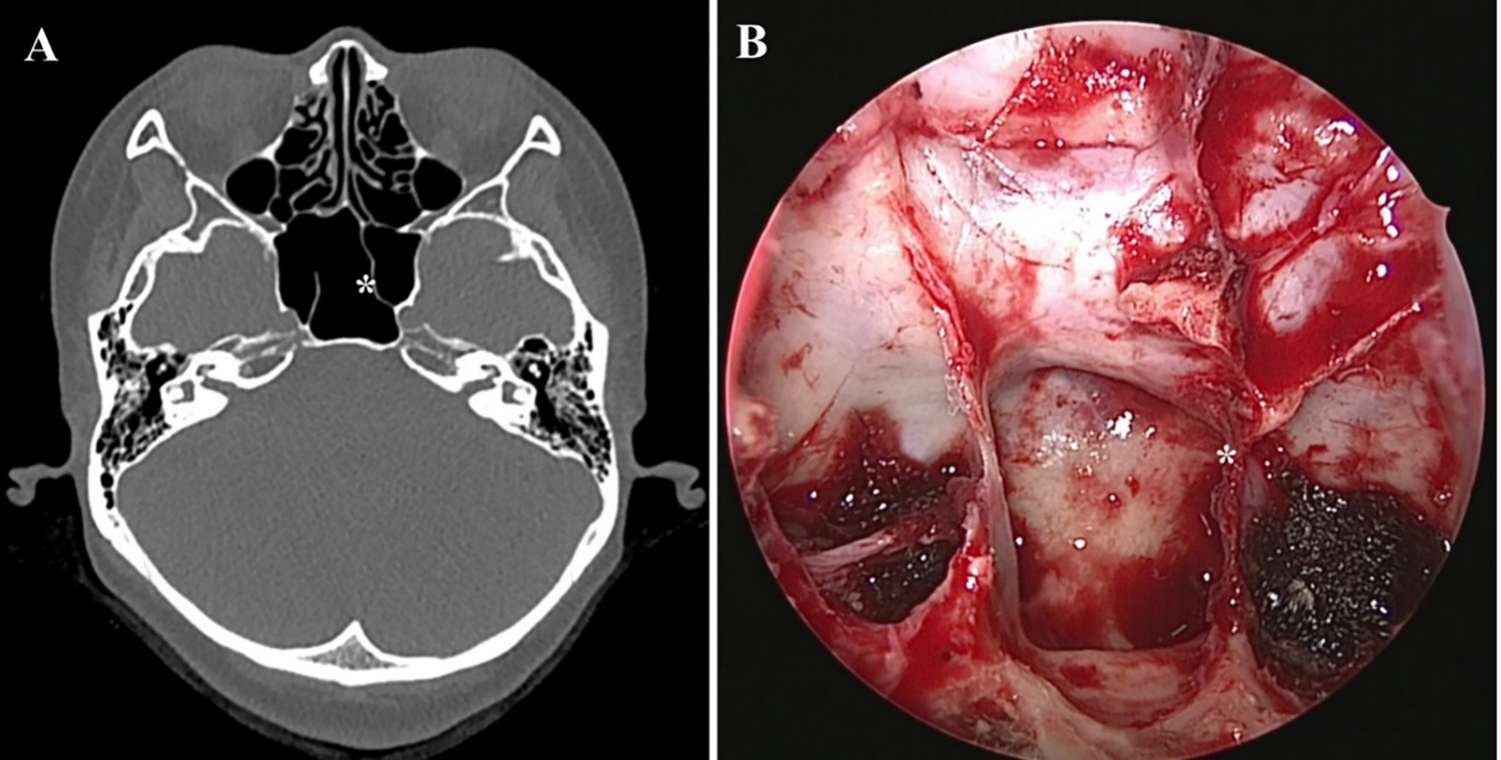
An intersphenoid sinus septum (asterisk) inserted into the paraclival internal carotid artery in a patient with postsellar pneumatization, as revealed by preoperative paranasal sinus computed tomography (A) and during endoscopic endonasal transsphenoidal surgery (B).

**Fig 2:**
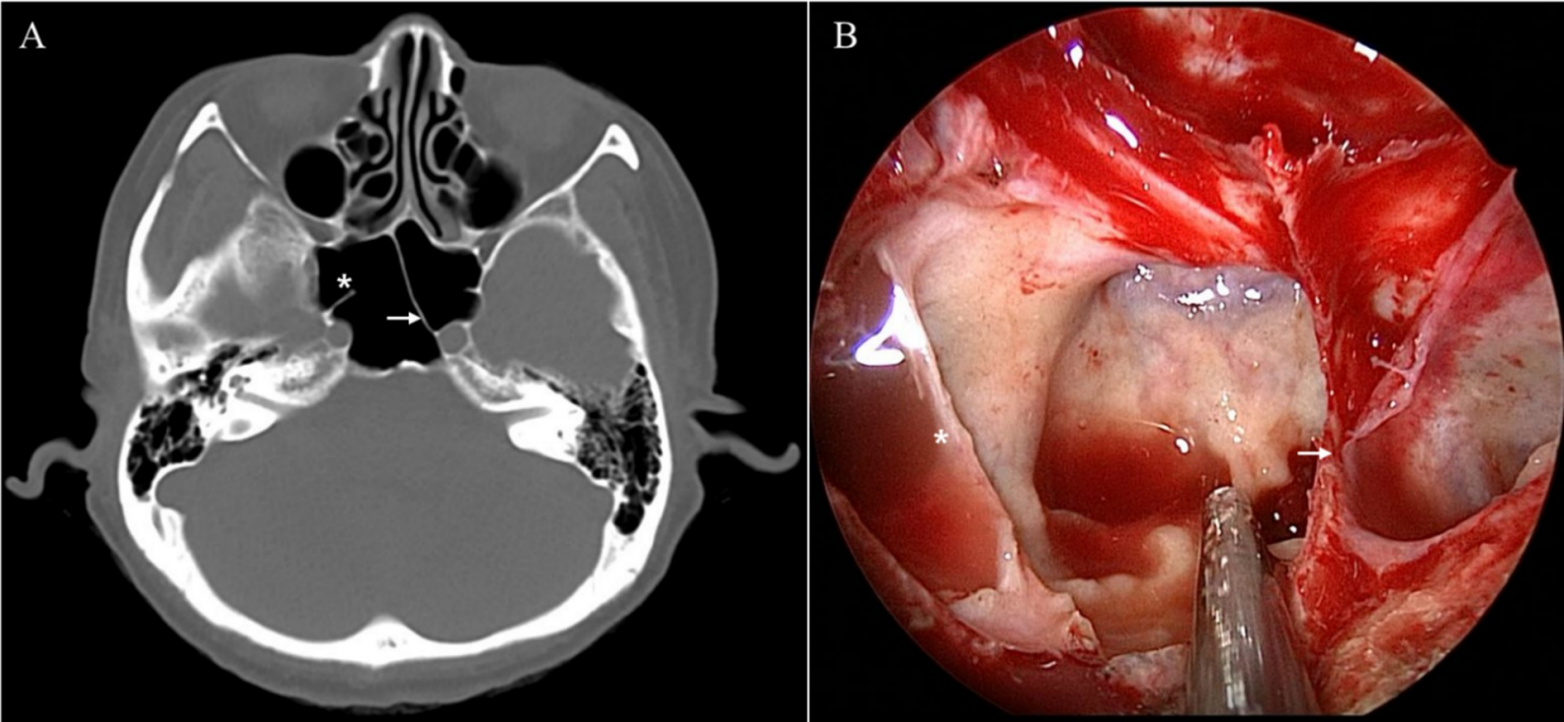
An intersphenoid sinus septum covering the paraclival internal carotid artery (asterisk) and entering that artery (arrow) in a patient with postsellar pneumatization, as revealed by preoperative paranasal sinus computed tomography (A) and during endoscopic endonasal transsphenoidal surgery (B).

**Fig 3:**
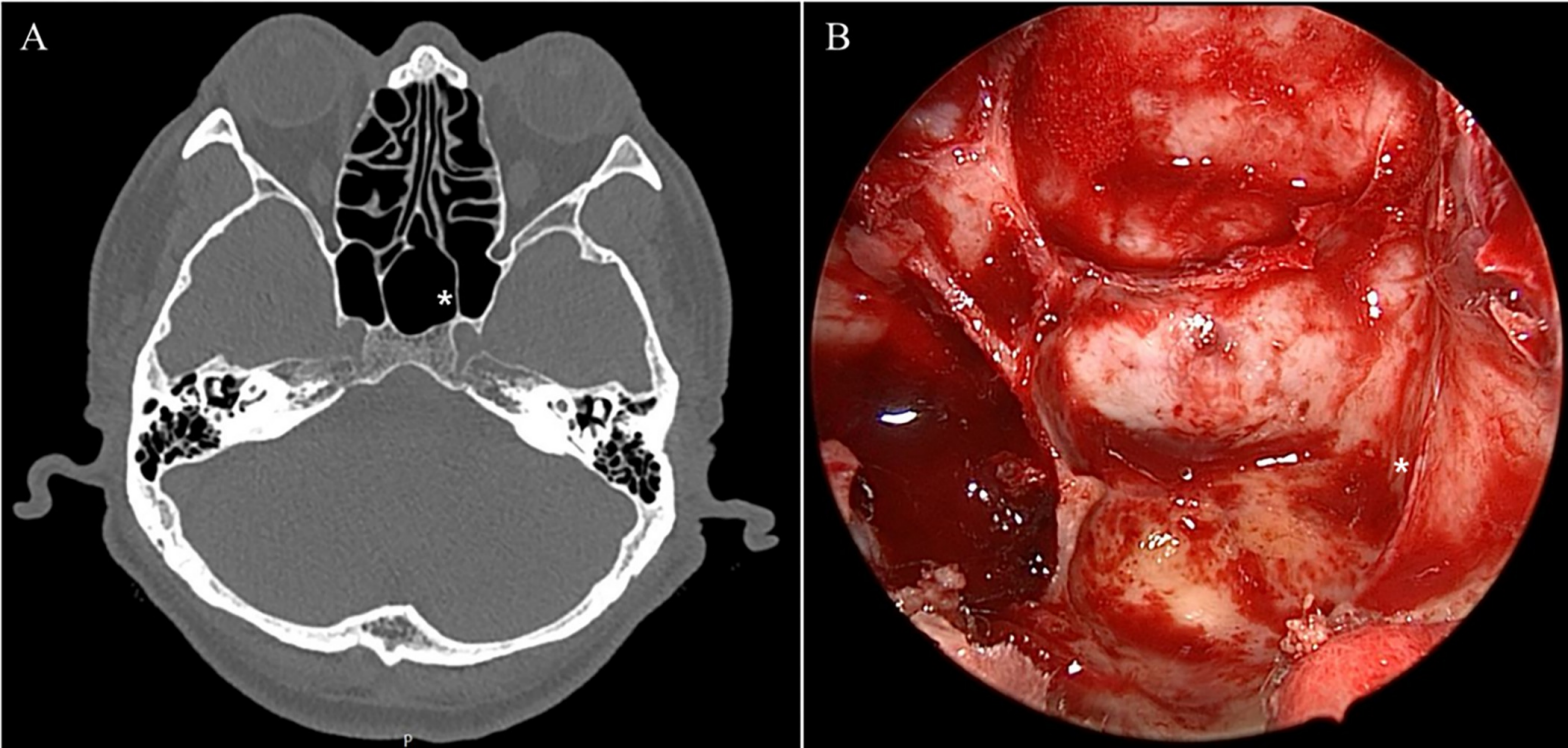
An intersphenoid sinus septum inserted into the paraclival internal carotid artery (asterisk) in a patient with presellar pneumatization, as revealed by paranasal sinus computed tomography (A) and during endoscopic endonasal transsphenoidal surgery (B).

During endoscopic endonasal transsphenoidal surgery, it is important to carefully check anatomical landmarks when approaching the sellar floor [20,21]. We thus examined the relationship between the ISS and ICA according to the extent of sphenoid sinus pneumatization (conchal, sellar, presellar, and postsellar types [22]). In previous studies, the postsellar type was predominant (46.7–86%) [1,23,24]; this was followed by the sellar type (29.4–98.8%) [5,9,21,25–28]. Our findings were similar: the postsellar type was present in 47.9% of patients, while the sellar type was present in 45.7% of patients. During endoscopic endonasal transsphenoidal surgery, ISS running from the posterior to the anterior wall in the center of the sphenoid sinus are clinically significant. The ISS may enter the paraclival ICA in patients with postsellar (Figs 1 and 2) or presellar (Fig 3) pneumatizations; it may cover the ICA in patients with postsellar pneumatizations (Fig 2). Accessory septa are present in 11.6–78.1% of patients [1–3,5,21,23,25–27]. Most studies have found that two ISS are common [2,29,30]. Sphenoid sinus aeration has been associated with sinus volume and ISS number [2,31].

In this study, aeration of the paraclival area was reduced and the ICA protruded to a lesser extent in patients with conchal or presellar pneumatizations than in patients with postsellar pneumatizations; this is presumably because better aeration leads to thinner bone between the ICA and the sellar floor. Although different criteria have been used to judge ICA protrusion, most papers have reported protrusion rates of 3.9% to 41% [2,14,30,32]. We found that presellar pneumatization tended to be more common when the bone around the ICA was thick, although this trend was not statistically significant. When the sphenoid sinus was well-aerated, the bone between the ICA and the sellae tended to be thin, but further studies are needed to confirm this finding.

The ISS insertion frequency into the ICA ranges from 8% to 89% [1,32–35]; our findings were within this range. We explored ISS and ICA locations, as well as the distance between them. In all patients, the ISS lay lateral to the paraclival ICA; however, this location was not always observed. As aeration increased, the distance from the ISS to the paraclival ICA tended to increase.

In summary, with increasing sphenoid sinus aeration, more ISS form; additionally, the paraclival ICA becomes more prominent and the distance between the ISS origin and the ICA increases. ICA prominence was strongly correlated with the thickness of bone surrounding the ICA, as well as ISS insertion into the ICA. Neither bone thickness nor ICA ISS insertion was significantly correlated with sphenoid sinus aeration. When aeration is good, the sinus walls are thin, while the opticocarotid recess and the carotid and optic grooves are visible. Thus, sellar floor access prior to central skull base surgery is easy [1,25]. In contrast, if the sphenoid sinus is poorly aerated, the sinus structures are unclear; extensive bone work is required, and the sinus septa are often attached to or lie around the ICA.

Our study had some limitations. First, most patients underwent surgery at a single center and had similar ethnicity; the relevant anatomy may vary according to ethnicity. This could make the risk of bias or ambiguity high. Second, lesions can deform normal anatomical structures. However, in actual endoscopic endonasal transsphenoidal surgery, it should be considered that most of the cases have lesions. We performed a PNS CT review to confirm and exclude cases with destroyed sphenoid sinus structure due to large sellar lesion.

## Conclusion

Patients with presellar pneumatization exhibit reduced ICA prominence, such that it is difficult to identify the ICA during endoscopic endonasal transsphenoidal surgery; the ISS is more likely to enter the ICA in such patients than in other patients. Preoperative PNS CT images should be used to check the tumor, as well as nearby anatomical structures and sphenoid sinus aeration status.
